# Direct comparison of ^99m^Tc-PSMA SPECT/CT and ^68^Ga-PSMA PET/CT in patients with prostate cancer

**DOI:** 10.22038/aojnmb.2019.43943.1293

**Published:** 2020

**Authors:** Batool Albalooshi, Mouza Al sharhan, Fariborz Bagheri, Shabna Miyanath, Bhavna Ray, Muhammed Muhasin, Seyed Rasoul Zakavi

**Affiliations:** 1 Dubai Nuclear Medicine and Molecular imaging Center-Dubai Health Authority, Dubai, UAE; 2 Department of Genetics and Pathology-Dubai Health Authority, Dubai, UAE; 3 Department of urology-Dubai Health Authority, Dubai, UAE; 4 Nuclear Medicine Research Center, Mashhad University of Medical Sciences, Mashhad, Iran

**Keywords:** Prostate Cancer, Tc-99m-PSMA, Ga-68-PSMA

## Abstract

**Objective(s):**

^99m^Tc-PSMA SPECT/CT is a cost effective alternative for ^68^Ga-PSMA PET/CT. The aim of this study was to directly compare these two techniques in patients with prostate cancer.

**Methods:**

28 man with prostate cancer were studied using ^99m^Tc-PSMA SPECT/CT and ^68^Ga-PSMA PET/CT in a short time period (<60 days). No intervention was done between the studies. Whole body PET/CT was done 60 minutes after IV injection of 2 MBq/Kg of ^68^Ga-PSMA. ^99m^Tc-PSMA kit (PSMA I+S) was used for SPECT/CT and whole body imaging was performed 4 hours after IV injection of 740 MBq of ^99m^Tc-PSMA. Images were interpreted independently and the results of each imaging were recorded.

**Results:**

The mean age of the patients was 64.7±9.6 years old and the mean time difference between two sets of images was 16.6±13.5 days. Abnormal uptake was seen in 25 (89.2%) patients by ^68^Ga-PSMA PET/CT and 20 (71.4%) patients with ^99m^Tc-PSMA SPECT/CT. No patients with positive ^99m^Tc-PSMA SPECT/CT had negative ^68^Ga-PSMA PET/CT. The mean number of detected lesions was 26.07±27.5 by ^68^Ga-PSMA PET/CT and 10.52±10.99 by ^99m^Tc-PSMA SPECT/CT (P<0.001). Detection of lymph nodes and bone metastases were not significantly different between two sets of imaging (P>0.05), however ^68^Ga-PSMA PET/CT were more successful in detection of prostate bed lesions compared to ^99m^Tc-PSMA scan. Interestingly, no patient with PSA level of >2.1 ng/ml had discordant result between two sets of images.

**Conclusion:**

^99m^Tc-PSMA SPECT/CT is as accurate as ^68^Ga-PSMA PET/CT in M staging, however ^68^Ga-PSMA PET/CT detected more lesions compared to ^99m^Tc-PSMA SPECT/CT. Detection rate was not significantly different between two techniques in patients with PSA levels>2.1 ng/ml.

## Introduction

 Prostate cancer is the most common cancer diagnosis made in men. Despite its indolent course in majority of patients, it is the third-leading cause of cancer death in men throughout the world (1).

 Multiple treatment options are available for patients with prostate cancer according to their risk of disease which is usually determined by Gleason score, PSA level and imaging results (1).

 Traditionally imaging evaluation for patients with prostate cancer included ultrasonography, CT, MRI and whole body bone scan. From PET/CT options, considering indolent nature of the disease FDG-PET/CT was not remarkable and ^18^F-Choline and 

^68^Ga-PSMA showed more sensitivity for detection of recurrence or metastases (2, 3). Radiolabeled prostate specific membrane antigen (PSMA) imaging of prostate cancer has been increasingly used in the last few years with promising results (3). Furthermore, labeling of PSMA with beta and alpha emitters provided a new option in treatment of patients with prostate cancer (4, 5). 

 PSMA is expressed in different tissues and organs including prostate, kidney, proximal small intestine and salivary glands and more importantly is overexpressed in high risk prostate cancer tissues (6, 7). Its overexpression in prostate cancer gives a promising outcome in imaging and treatment of patients with prostate cancer (7). 

 Although ^68^Ga-PSMA has been widely studied, ^99m^Tc-PSMA is introduced as a cost effective alternative either for imaging or for radio-guided surgery (8). Wide availability of SPECT/CT machines facilitated accurate localization of the lesions (9). 

 However there are limited studies comparing ^68^Ga-PSMA PET/CT scan with ^99m^Tc-PSMA SPECT/CT in terms of sensitivity, specificity and accuracy (10-13). The aim of this study was performing a head to head comparison of ^68^Ga-PSMA PET/CT scan with ^99m^Tc-PSMA SPECT/CT in a group of patients with prostate adenocarcinoma who referred either for initial staging, restaging or recurrence evaluation.

## Methods

 We studied 28 men with prostate carcinoma who referred to our center for PSMA imaging. The indications for imaging were recurrence evaluation or restaging in 24 patients and initial staging in 4 patients. All patients were explained about the procedures and probable use of data for research project and signed a written consent. The study were confirmed by local ethics committee (DSREC-02/2019_20). All patients underwent ^68^Ga-PSMA PET/CT and ^99m^Tc-PSMA SPECT/CT in a short time interval (<60 days). Whole body PET/CT was done using GE MI-DR( 64 SLICE CT) and patients studied 60 minutes after IV injection of 2 MBq/Kg of ^68^Ga-PSMA (8 beds and 4 minutes per bed position, CT: 120 Kvp, auto mA (range 30 to 300 mA) with 40 % dose reduction and noise index 16.0. 

^99m^Tc-PSMA kit (PSMA I+S) was labeled with ^99m^Tc-pertechnetate and whole body imaging was performed 4 hrs after IV injection of 740 MBq of ^99m^Tc-PSMA (Exposure time per pixel: 480 sec and pallet velocity: 5.0 cm/min, stored in a 1024×256 matrix). SPECT was done with step and shoot protocol, 30 sec/view over 360 degree and stored in a 128×128 matrix.

 The CT part of the SPECT/CT was done according to the following protocol: helical, 120kvp, smart mA (50-150 mA) Noise index: 18.4752, slice thickness: 3.75 mm.

 Images were reviewed separately and anonymously by two experienced nuclear physicians and were categorized as normal or abnormal. Any non-physiologic uptake was considered as abnormal finding. Any suspicious finding was further evaluated by delayed imaging and post void images. In case of discrepancy between readers, consensus was obtained by consulting with a third expert. Furthermore, the locations of abnormal uptake were fully described in both sets of images with special attention on prostate bed, local or regional lymph nodes and skeletal system. 

***Statistical analysis:***

 Statistical analysis was done using SPSS software (Version 16, SPSS Inc. USA). Frequency and cross tables were used for descriptive analysis of the variables. Comparison of quantitative and qualitative variables between two sets of images was done using paired t-test and McNemar test respectively. P<0.05 was considered statistically significant in all comparisons. 

## Results

 We studied 28 men with prostate cancer with mean age of 64.7±9.6 years old and age range of 49 to 90 years old. Histopathologic analysis revealed classic type adenocarcinoma in all patients with evidence of poor differentiation in one patient. The reason for referral was restaging in 16 patients (57.1%), recurrence evaluation in 8 (28.6%) and initial staging in 4 (14.3%) patients. The median Gleason score was 7. Serum PSA level ranged from 0.03 to 438 ng/ml with a mean of 54.8±97.0. All patients underwent ^68^Ga-PSMA and ^99m^Tc-PSMA whole body imaging. The time difference between two sets of imaging ranged between 2 to 50 days with a mean of 16.6±13.5 days.

^68^Ga-PSMA scan showed abnormal uptake in 25 patients while ^99m^Tc-PSMA scintigraphy found abnormal uptake in 20 patients. Three patients had negative finding in both sets of images. Five patients had negative ^99m^Tc-PSMA scintigraphy while ^68^Ga-PSMA scan was abnormal (table 1). All patients with abnormal ^99m^Tc-PSMA scan had abnormal findings on ^68^Ga-PSMA imaging too. The ^68^Ga-PSMA scan seems to be more sensitive than ^99m^Tc-PSMA scan in detection of patients with abnormality (P=0.06). 

**Table 1:** Comparison of overall finding of ^99m^Tc-PSMA scan versus ^68^Ga-PSMA scan (McNemar test; P=0.06)

		^99m^ **Tc-PSMA scan**		**T** **otal**
		**Negative**	**Positive**	
^68^Ga-PSMA scan	Negative	3	0	3
	Positive	5	20	25
Total		8	20	28

 Further analysis was done for lesion detection in prostate bed, lymph nodes and skeletal system. Abnormal uptake in prostate bed was seen in 24 patients by ^68^Ga-PSMA scan which was much higher than 15 patients with abnormal prostate bed uptake in ^99m^Tc-PSMA scan (P=0.004). Table 2 shows abnormal uptake in prostate bed in two sets of images.

**Table 2:** Prostate bed lesion in ^99m^Tc-PSMA scan versus ^68^Ga-PSMA scan. (McNemar test: P=0.004)

		^99m^ **Tc-PSMA scan**		**Total**
		**Negative**	**Positive**	
^68^Ga-PSMA scan	Negative	4	0	4
	Positive	9	15	24
Total		13	15	28

We found a remarkable agreement between ^99m^Tc-PSMA scan and ^68^Ga-PSMA scan for detection of lymph node metastasis (Kappa coefficient=0.92). Anyhow one patient had no lymph node metastasis in ^99m^Tc-PSMA scan while ^68^Ga-PSMA scan detected a lymph node metastasis. Table 3 shows no significant difference between two sets of images in terms of detection of lymph node metastasis (McNemar Test; P=1.0).

**Table 3:** Lymph node metastasis in ^99m^Tc-PSMA scan versus ^68^Ga-PSMA scan (P=1.0)

		^99m^ **Tc-PSMA scan**		**Total**
		**Negative**	**Positive**	
^68^Ga-PSMA scan	Negative	16	0	16
	Positive	1	11	12
Total		17	11	28

Regarding detection of bone metastasis, complete agreement was noted between two sets of images. All patients with bone metastases was detected by both methods (P=1.0). 

 Overall ^68^Ga-PSMA scan detected more abnormal foci compared to ^99m^Tc-PSMA scan (P=0.017). The number of detected lesions ranged from 0 to 65 in ^68^Ga-PSMA scan while it ranged from 0 to 30 in ^99m^Tc-PSMA scan. Using paired t-test, the mean number of lesions was compared between two set of images. It was significantly higher in ^68^Ga-PSMA scan (26.07±27.5) compared to ^99m^Tc-PSMA (10.52±10.99) scintigraphy (P<0.001). 

 Five patients had discordant scan results between ^68^Ga-PSMA scan and ^99m^Tc-PSMA scan that was further analyzed. PSA level ranged from 0.03 to 2.1 ng/ml in these patients and the mean PSA level was 0.63±0.85 ng/ml which was significantly lower than mean PSA level of 66.5±103.6 ng/ml in patients with concordant results. No patient with PSA >2.1 ng/ml had discordant results. None of the patients with discordant results had more than 1 lesion in ^68^Ga-PSMA scan. The mean age, mean time interval between two imaging and mean Gleason score was not significantly different between patients with discordant results and those with concordant results (P>0.3). 

 Defining a PSA threshold of more than 0.5 ng/ml, there was no statistically significant difference between two sets of scans in detection of abnormality. In these patients, concordance was noted in 21 out of 23 patients between the two sets of images while 2 patients had positive ^68^Ga-PSMA scan and negative ^99m^Tc-PSMA scan (P=0.50). There was no significant difference between the two methods in detection of lymph node metastasis (P=1). Anyhow the number of lesions in ^68^Ga-PSMA scan was higher compared to ^99m^Tc-PSMA scan, independent of PSA level.

## Discussion

 Overall we found that ^68^ Ga-PSMA PET/CT seems to be more sensitive than ^99m^Tc-PSMA SPECT/CT in terms of detection of lesions, however this superiority is not evident in detection of bone metastases.

 Bone metastasis is the most common distant metastasis in patients with prostate cancer and bone scan using ^99m^Tc-HDP is usually used for detection of it , however false positive results are common due to HDP uptake in variety of benign boney lesions and the overall specificity is low  (14) . Lengana et al observed the superiority of ^68^Ga-PSMA PET/CT over bone scan in detecting lytic and bone marrow lesions and suggested it as a better alternative to replace the bone scan (15) . In another study, ^99m^Tc-PSMA SPECT/CT imaging found 95 bone metastases in 25 patients which was superior to other imaging modalities such as bone scan and MRI  (16) .

 Considering equal sensitivity of ^68^Ga-PSMA PET/CT and ^99m^Tc-PSMA SPECT/CT in our study for detection of bone metastasis, we may expect superior sensitivity of ^99m^Tc-PSMA SPECT/CT over ^99m^Tc-HDP bone scan too, although further studies with direct comparison is needed to confirm the findings.

 The use of ^68^Ga-PSMA PET/CT in initial staging of prostate cancer can make a significant impact on therapy. It was more sensitive and specific in detection of prostate cancer compared to current conventional imaging modalities  (17) .

 Additionally a study group from Munich showed that even at low PSA levels of 1 to >2 ng/ml, ^68^Ga-PSMA PET/CT could detect 93% of lesions  (18) . A strong association has been shown between increased PSA levels and metastatic detection using ^68^Ga-PSMA PET/CT in many studies  (16,18, 19) . Natarajan et al showed that the detection rate was higher for higher PSA levels using ^68^Ga-PSMA PET/CT i.e: 86%, 85% and 95% for PSA levels of 2-5, 1-10, and 10 ng/ml respectively  (20) . In another study with ^99m ^Tc-PSMA-SPECT-CT, the detection rate according to PSA levels were 30%, 80% and 100% for PSA levels of >1, 4-10, < 10 ng/ml respectively  (21) .

 Anyhow, joint SNM/EANM guideline on ^68^Ga-PSMA PET/CT recommends it for localization of the recurrence in patients with PSA levels of 0.1-10 ng/ml  (22) . Our study showed that ^99m^Tc-PSMA SPECT/CT has no significant difference with ^68^Ga -PSMA PET/CT in localization of recurrence in patients with PSA levels of more than 2.1 ng/ml.

 Considering limited availability and high cost of ^68^Ga-PSMA PET/CT, prostate cancer imaging with ^99m^Tc-PSMA SPECT/CT should be considered as a good alternative. We checked the costs of performing ^68^Ga-PSMA PET/CT and ^99m^Tc-PSMA SPECT/CT in few countries in the middle East and found that ^68^Ga-PSMA PET/CT is 2.4 to 7.7 times more expensive than ^99m^Tc-PSMA SPECT/CT. Our findings showed that using ^99m^Tc-PSMA SPECT/CT in management of patients with prostate cancer, may have significant impact on health economics of prostate cancer in the developing countries.. 

**Figure 1:**
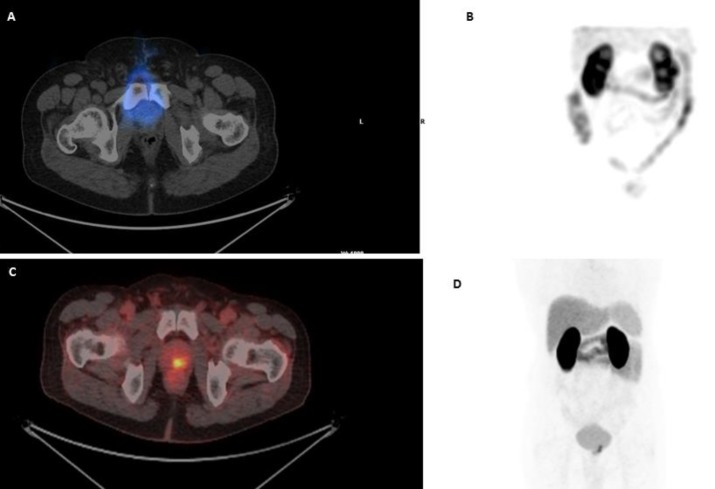
^99m^Tc-PSMA SPECT/CT transaxial view (A) of the pelvis and planar anterior view (B) shows no PSMA uptake in the prostate gland. ^68^Ga-PSMA PET/CT fused images (C) and whole body scan (D) shows focal small PSMA avidity in the prostate gland

 Our study showed that ^99m^Tc-PSMA scan is as sensitive as ^68^Ga-PSMA scan in patients with prostate cancer in terms of detection of abnormality, lymph node metastasis or skeletal involvement, however it is less sensitive in detection of lesions in prostatic bed (Figure 1).

Our results are concordant with the results of a joint study from South Africa, Netherland and Belgium which showed that ^99m^Tc-PSMA scan might be valuable in imaging of prostate malignancy although with poor efficiency in small sized lesions and its utilization was not suggested in patients with small volume disease. In the same study all lymph nodes greater than 10 mm were detected while only 28% of the lymph nodes less than 10 mm were detected by ^99m^Tc-PSMA scan  (10) .

 In another study head to head comparison of qualitative and semi-quantitative performance of ^99m^Tc-EDDA/HYNIC-iPSMA SPECT/CT and ^68^Ga-PSMA-11 PET/CT was done in 23 patients and the published data were suggestive of comparable findings between the two tracers supporting the use of ^99m^Tc-PSMA SPECT/CT in patients with progressive metastatic castration-resistant prostate cancer (11) . 

**Figure 2:**
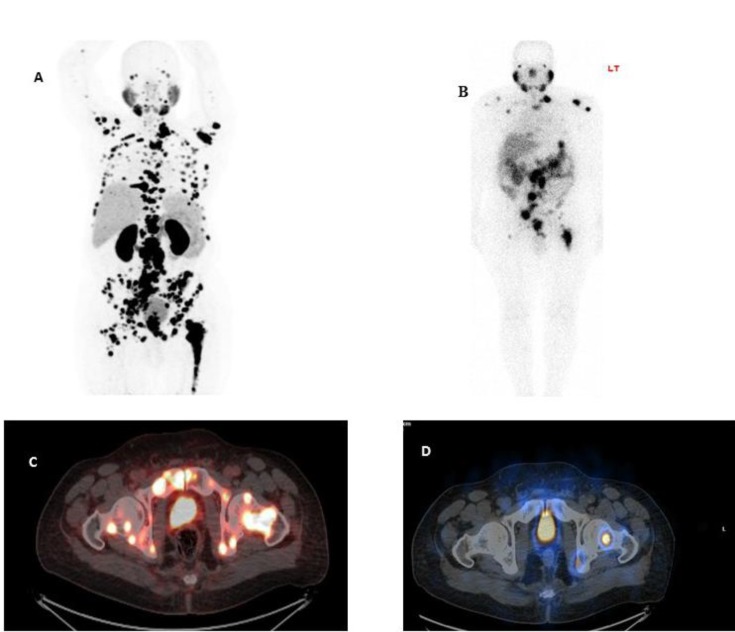
^68^ Ga-PSMA PET-MIP image (A) and fused PET/CT image of the pelvic region (C) shows extensive multiple bone and lymphatic metastases. ^99m^ Tc-PSMA anterior planar whole body (B) and corresponding SPECT/CT slice (D) shows fewer PSMA avid lesions

 Our study, however showed that the mean number of detected lesions in ^99m^Tc-PSMA scan were significantly less than ^68^Ga-PSMA scan (10.52±10.99 vs 26.07±27.5 respectively) (Figure 2). 

 Lawal et al demonstrated superiority of ^68^Ga-PSMA versus ^99m^Tc-PSMA in terms of number of detected lesions and in the published data accordingly 46 lesions were detected in ^68^Ga-PSMA while ^99m^Tc-PSMA SPECT-CT detected only 36 lesions which were localized to the prostate, lymph nodes and bones  (10) . 

 In our study, although the number of detected lesions was higher in ^68^Ga-PSMA PET/CT compared to ^99m^Tc-PSMA SPECT/CT, there was no statistically significant difference in terms of localization of abnormality in patients with PSA level of >0.5ng/ml. Therefore ^99m^Tc-PSMA imaging offers a good alternative in the absence of ^68^Ga-PSMA PET Imaging and it is also cost effective enabling many centers to proceed with prostate cancer imaging work up. ^99m^Tc-PSMA SPECT/CT may also be considered in selection of patients suitable for ^177^Lu-PSMA therapy and radio guided surgery  (8) .

## Conclusion

^99m^Tc-PSMA SPECT/CT is a cost effective alternative to ^68^Ga-PSMA PET/CT with no significant difference in M staging. The mean number of detected lesions was significantly higher in ^68^Ga-PSMA PET/CT compared to ^99m^Tc-PSMA SPECT/CT. Although ^68^Ga-PSMA PET/CT was more successful in detection of prostate bed lesions compared to ^99m^Tc-PSMA scan, detection of lymph nodes and bone metastases was not significantly different. Finally, no patient with PSA of >2.1 ng/ml had discordant result between two sets of images. 
